# Coiled Coil Rich Proteins (Ccrp) Influence Molecular Pathogenicity of *Helicobacter pylori*

**DOI:** 10.1371/journal.pone.0121463

**Published:** 2015-03-30

**Authors:** Sarah Schätzle, Mara Specht, Barbara Waidner

**Affiliations:** 1 Department of Medical Microbiology and Hygiene, Institute of Medical Microbiology and Hygiene, University Hospital Freiburg, Hermann-Herder Straße 11, 79104 Freiburg, Germany; 2 Department of Microbiology, Faculty for Biology, University of Freiburg, Schaenzle Straße 1, 79104 Freiburg, Germany; 3 LOEWE Center for Synthetic Microbiology, Hans-Meerwein Straße 35032 Marburg, Germany; University of Illinois at Chicago College of Medicine, UNITED STATES

## Abstract

Pathogenicity of the human pathogen *Helicobacter pylori* relies on its capacity to adapt to a hostile environment and to escape the host response. Although there have been great advances in our understanding of the bacterial cytoskeleton, major gaps remain in our knowledge of its contribution to virulence. In this study we have explored the influence of coiled coil rich proteins (Ccrp) cytoskeletal elements on pathogenicity factors of *H*. *pylori*. Deletion of any of the *ccrp* resulted in a strongly decreased activity of the main pathogenicity factor urease. We further investigated their role using *in vitro* co-culture experiments with the human gastric adenocarcinoma cell line AGS modeling *H*. *pylori* - host cell interactions. Intriguingly, host cell showed only a weak “scattering/hummingbird” phenotype, in which host cells are transformed from a uniform polygonal shape into a severely elongated state characterized by the formation of needle-like projections, after co-incubation with any *ccrp* deletion mutant. Furthermore, co-incubation with the *ccrp59* mutant resulted in reduced type IV secretion system associated activities, e.g. IL-8 production and CagA translocation/phosphorylation. Thus, in addition to their role in maintaining the helical cell shape of *H*. *pylori* Ccrp proteins influence many cellular processes and are thereby crucial for the virulence of this human pathogen.

## Introduction

*Helicobacter pylori* is a Gram-negative, microaerophilic, helical-shaped, flagellated bacterium that colonizes the gastric mucosa of humans [1]. It is the etiological microbial agent of chronic gastritis and peptic ulcers [2] and a risk factor for gastric adenocarcinoma [3] and B-cell MALT lymphoma [4]. The clinical outcome of *H*. *pylori* infection is determined by the genetic predisposition of the host as well as by environmental and bacterial factors. As such, *H*. *pylori* produces numerous virulence factors [5] that enable the bacteria to adapt to and multiply within the hostile environment of the human gastrointestinal tract [6].

One of the main pathogenicity factors of *H*. *pylori* is the urease enzyme which helps the bacteria to withstand the acidic pH by hydrolyzing urea into carbon dioxide and ammonia. The enzyme activity is essential for both early colonization events and for virulence [7,8]. *H*. *pylori* urease is produced in large amounts, accounting for up to 10% of total cellular proteins [9]. It was shown to form a giant 1.1 MDa complex containing 12 subunits of UreA and UreB, with two Ni^2+^ needed for enzyme activity and a complex, timely ordered assembly process [10,11]. Apart from its role in the successful colonization of *H*. *pylori*, urease might also indirectly interfere with host cell functions.

Another outstanding virulence determinant is the *cag*-type IV secretion system (*cag*-T4SS) since its presence in the bacterial genome strongly correlates with serious *H*. *pylori-*induced pathologies [6]. This system is encoded on the *cag*-pathogenicity island (*cag*-PAI) [12], which represents one of the major variable genome regions of *H*. *pylori*. The effector protein CagA (cytotoxin-associated gene A) as well as peptidoglycan [13] are injected into the host cell resulting in an activation of nuclear factor (NF)-κB and gastric inflammation. Some of these *cag*-T4SS components such as CagT are essential for translocation of the effector protein [14] while others like CagY alter the function of the T4SS and seem to modulate the host immune response to promote bacterial persistence [15]. Translocated CagA is then phosphorylated by host cell kinases at tyrosine residues contained within Glu-Pro-Ile-Tyr-Ala (EPIYA) motifs in the C-terminal region of the protein [12,16,17]. Both phosphorylated and non-phosphorylated forms of CagA interact with host cell signaling proteins resulting in an assortment of consequences including cytoskeletal alterations that lead to the “hummingbird” cell phenotype [18,19], disruption of cellular junctions and altered cellular adhesion and polarity [17,20,21]. The “needle”-like structure of the *cag*-T4SS interacts with the integrin α5β1-receptor on the gastric epithelial cells to deliver CagA into the host cells [22], which leads to an activation of signaling cascades inducing pro-inflammatory cytokines like IL-8 [23,24]. Yet another virulence factor of *H*. *pylori* is the helical cell shape accounting for the corkscrew-like motion of the bacterium, which enables the pathogen to penetrate into and to move within the viscous mucus layer and provides protection from peristalsis and luminal acidity [25]. Recent research revealed that the cell shape of *H*. *pylori* is apparently controlled by two unrelated mechanisms that operate at different levels: peptidases influence cell shape by causing peptidoglycan relaxation [26,27], whereas we demonstrated that so-called *coiled-coil-rich proteins* (Ccrp) influence cell shape most probably by composing an intracellular scaffold [28],[29]. Remarkably, mutants of *H*. *pylori* lacking a cell wall tripeptide protease displayed a rod shaped phenotype and were attenuated in stomach colonization without apparent changes in proinflammatory activity [26]. Ccrp proteins have a molecular architecture which is reminiscent of that of intermediate filaments [30,31]. *H*. *pylori* contains four Ccrps (Ccrp58, Ccrp59, Ccrp1143, and Ccrp1142) spontaneously polymerizing in the absence of any cofactor *in vitro*. Deletion of *ccrp59* results in the complete loss of helical cell shape, while inactivation of other *ccrp* genes affects cell morphology to a lesser extent depending on the strain background. Additionally, all four *ccrp* mutants significantly impair motility despite of apparently unaltered flagella morphology [29]. However nothing is known about the influence of Ccrps on pathogenicity. Due to their analogy to intermediate filaments we assumed a role of these proteins in cell stability which might in turn have an impact on membrane protein composition and thereby on the spatial organization e.g. of virulence factors like proteins of the *cag*-T4SS. We therefore explored whether deletion of Ccrp proteins influences molecular pathogenicity in *H*. *pylori*. Strikingly, we found that loss of any of the *ccrp* genes resulted in a strongly decreased activity of the main pathogenicity factor urease. Also, in *in vitro* co-culture experiments modeling *H*. *pylori—*host cell interactions, these mutants were unable to induce comparable levels of the “hummingbird” phenotype of AGS cells seen with wild-type *H*. *pylori*. Furthermore, the *ccrp59* mutant resulted in greatly reduced *cag*-T4SS associated activities like IL-8 production and CagA translocation/phosphorylation.

## Material and Methods

### Bacteria and cell culture

Bacterial strains are listed in Table 1. *H*. *pylori* strains were routinely cultivated on Dent blood agar in a microaerobic atmosphere as described earlier [32]. Growth experiments were performed in Brucella broth with 5% (v/v) fetal calf serum (BBF). Infection experiments were performed in Hams F-12 medium supplemented with L-glutamine and 5% fetal bovine serum. Growth rate was assessed by optical density (OD_600_).

**Table 1:** Strains, plasmids and primers used in this study.

**Strain, plasmid, primer**	**Relevant characteristics**	**Source**
**Strains**		
***E*.*coli***		
DH5α	F^-^, φ80d*lacZ*ΔM15, Δ(*lacZYA-argF*)U169, *deoR*, *recA*1, *endA*1, *hsdR*17(rk^-^, mk^+^), *phoA*, *supE*44, λ^-^, *thi*-1, *gyrA*96, *relA*1	Bethesda Research Laboratories
***H*. *pylori***		
G27	wt, clinical isolate	
G27ΔCagE	G27, *cagE*::*cat*	A. Covacci
G27-59CAT	G27, *ccrp59*::*cat*	[28]
KE88-3887	piglet-passaged strain 26695	[72]
KE-58PCAT	KE, *ccrp58*::*PCAT*	[29]
KE-59PCAT	KE, *ccrp59*::*PCAT*	[28]
KE-1142PCAT	KE, *ccrp1142*::*PCAT*	[29]
KE-1143PNEO	KE, *ccrp1143*::*PNEO*	[28]
KE- DKO59-1143	KE, *ccrp59*::*PCAT*, *ccrp1143*::*PNEO*	[28]
KE-VKO	KE, *ccrp58*, *ccrp59*::*PCAT*, *ccrp1142*,*ccrp1143*::*PNEO*	This study
KE-59PCAT-K	KE, *ccrp59*::*PCAT*, *cagA*::*Ppfr-ccrp59*	This study
KE-59PCAT-K2	KE, *ccrp59*::*PCAT*, *rdxA*::*Pccrp59—ccrp59*	This study
**Plasmids**		
pTn*Max*5	*lac*I^q^, *tnp*R, *tnp*A, *res*, *ori*_fd_, *cat*_GC_, Cm^R^	[73]
pZERO-2	Cloning vector, MCS in *lacZ'*, *neo*, Km^r^	Invitrogen
pZERO-1	Cloning vector, MCS in *lacZ'*, *zeo*, Zeo^r^	Invitrogen
p58-59-PCAT	pZERO-2, Δ HP0058-0059::*Pcat*, Cm^r^, Km^r^	This study
p1142-1143-PNEO	pZERO-1, Δ HP1142-1143::*Pneot*, Zeo^r^, Km^r^	This study
pSLpPFR59KM	pSL-P_*pfr*_1021 cm containing *ccrp59* and a kanamycin resistance cassette instead of chloramphenicol	This study
p*ureAB*-PCAT	pZERO-2, Δ *ureAB*::*Pcat*, Cm^r^, Km^r^	This study
pRDX-K-Pccrp59	pRDX-C-P_*ccrp59*_ *ccrp59* containing *ccrp59 with its own promoter* and a kanamycin resistance cassette instead of chloramphenicol	This study
**Primer**		
0057-L1	GCGGTAATTAATTCTCATTC	This study
PCAT-KP 58-59-R1	GGCGGATTAACAAAAACCGGAAAGAATATCTCCTTTTATCT	This study
CAT-KP 58-59-L1	TGGCAGGGCGGGGCGTAATTAAAGGAGAAATATCATGG	This study
0060-R3	TTAGACAAGCTAGGCACATC	This study
UREB-L1	GGGGTTTCTAATGTTTTAGACCGG	This study
CATUREB-R1	CTCCTGAAAATCTCGGACTTATTCTCCTATTCTTAAAGTG	This study
CATUREB-L1	TGGCAGGGCGGGGCGTAAGATTTTTTAGGAGCAACGCTCC	This study
UreB-R1	GCTACGAATAAGCTATACCAAG	This study
HP0059-5Kpn	gtgcctggtaccTCATAAAATGGGAACATTCATTGAA	This study
HP0059-3Pst	aaaccactgcagttatggttttggttgttttgaggg	This study
P-Ccrp59-L1 (BamHI)	GCAGGATCCGGTGGTCTTAATCGGCTATA	This study
CATS1	TCCGGTTTTTGTTAATCCGCC	This study
CATAS1	TTACGCCCCGCCCTGCCA	This study
CATS2	TCCGAGATTTTCAGGAG	This study
KMS1	AAAATTGGAACCGGTACG	This study
KMAS1	AGACATCTAAATCTAGG	This study

*E*. *coli* strains were grown aerobically at 37°C in Luria-Bertani medium. When appropriate, growth media were supplemented with 50 μg/ml ampicillin (Ap) or 20 μg/ml chloramphenicol (Cm).

Human gastric adenocarcinoma AGS cells (CRL-1739; ATCC) were cultured in Hams F-12 medium with L-glutamine (PAA) supplemented with 5% fetal bovine serum (Invitrogen) at 37°C in 5% CO_2_.

### Construction of *H*. *pylori* mutants

The isogenic *H*. *pylori* quadruple *ccrp* deletion mutants and the *ureAB* deletion mutant were constructed as described earlier [28,29]. Briefly, resistance marker genes (*cat*, *Pcat*, *Pneo*) were fused to upstream and downstream DNA regions of mutagenized genes by using a modified version of the megaprimer PCR protocol [33,34] and primers listed in Table 1. Subsequently marker exchange mutagenesis of *H*. *pylori* was performed according to standard procedures [35]. *H*. *pylori* mutants carrying the resistance genes inserted into the chromosome were selected by growth on Dent blood agar containing chloramphenicol or/and kanamycin at concentrations of 20 mg/l. The correct insertions were verified by PCR and sequencing. Complementation of KE-59PCAT was achieved by using the genetic complementation system according to Schär *et al*. [36,37]. Briefly, pSL-P_*pfr*_1021 cm [37] was digested with *Kpn*I-*Pst*I and successively ligated with the *Kpn*I-*Pst*I fragment encoding *ccrp59* amplified with primer pair HP0059-5KPN/HP0059-3PST (Table 1) *and the Pst*I fragment encoding the kanamycin resistance cassette from *Campylobacter coli* derived from pSL-1043::km2 [36]. Plasmid pSLPpfr59KM was then used for the transformation of *H*. *pylori* KE88-3887 which was performed as described [28]. The resulting *H*. *pylori* mutant was named KE-59PCAT-K. The second complementation of KE-59PCAT was achieved by using the genetic complementation system according to Croxen *et al*. [38]. Ccrp59 including its own promoter was amplified with primer pair P-Ccrp59-L1 BamHI /HP0059-3PST (Table 1), subsequently digested and successively ligated in pRDX-K, which is pRDX-C [38] modified with the kanamycin resistance cassette described in [36]. Plasmid pRDX-G-Pccrp59 was then used for the transformation of *H*. *pylori* KE88-3887 as described [28] and the resulting *H*. *pylori* mutant was named KE-59PCAT-K2.

Restriction and modifying enzymes (New England Biolabs, USA) were used according to the manufacturer’s instructions. Cloning was performed in *E*. *coli* according to standard protocols. Plasmids were isolated with a QIAprep Spin Miniprep Kit from Qiagen.

### Analysis of urease activity

Urease activity was determined in fresh lysates by measuring ammonia production from hydrolysis of urea, as described previously [32,39]. The concentration of ammonia in the samples was inferred from a standard NH_4_Cl concentration curve.

### Qualitative and quantitative analysis of the AGS cell hummingbird phenotype

Mid-logarithmic phase bacteria grown in Hams F-12 medium supplemented with L-glutamine and 5% fetal bovine serum were used for infection of AGS cells. The density of the bacteria was determined by optical density measurement. AGS cells (7 x 10^4^) cultured on glass cover slips (R. Langenbrinck) in 24-well plates were infected with either wild-type or mutant *H*. *pylori* (7 x 10^6^) at a MOI of 100 for 4 h. After a washing step with PBS, fixation with methanol for 10 minutes and three washing steps with PBS the cover slips were turned upside down on a slide with PermaFluor aqueous mounting medium (Thermo Scientific). The percentage of elongated cells was determined in a blinded manner to determine the numbers of cells having the hummingbird phenotype. Elongated cells were defined as reported by Backert *et al*. [40]. For each strain between 1830 and 2800 cells were counted and evaluated. Cells were inspected with a BZ-9000E (KEYENCE) microscope. Images were acquired with a monochromatic CCD camera driven by BZII Viewer (KEYENCE) software. All samples were examined in triplicate in at least three independent experiments. The data are presented as the means of three independent experiments.

### Bacterial adherence assay

*H*. *pylori* adherence to AGS cells was investigated using two different standard assays. For the first assay, AGS cells and *H*. *pylori* were grown as described above. AGS cells (4 x 10^6^) cultured in chamber slides (Nunc) were infected with either wild-type or mutant *H*. *pylori* at MOI of 1:100 for 24h. Cells were washed with PBS, fixed with 3.7% paraformaldehyde and blocked with blocking buffer (10% (v/v) fetal bovine serum, 4% (w/v) BSA in PBS buffer). Actin condensation was revealed by phalloidin-FITC staining (Sigma). The presence of *H*. *pylori* was demonstrated by additional staining with rabbit anti-*H*. *pylori* polyclonal antibodies (Biomeda Corp, Foster City, CA, USA) and anti-rabbit IgG Cy3 conjugate (Biomeda Corp, Foster City, CA, USA). Specimens were examined using an Axioplan 2 imaging microscope (Carl Zeiss) with AxioCam MRm camera and Axiovision 4.7.1 software (Carl Zeiss). Fluorescence images were collected and statistical analysis was done by counting adherent bacteria per AGS cell (using 600–1100 AGS cells per experiment).

### Investigation of viability and adhesion activity

*Helicobacter pylori* viability and adherence to AGS cells was investigated using a standard colony-forming assay [41]. In brief, AGS cells (2 x 10^5^ cells per well) grown in six-well plates were infected with the wild-type or different *H*. *pylori* cytoskeletal mutant suspension (4 x 10^6^ cfu) at an MOI of 20. The bacteria were centrifuged (500 g for 5 min) onto the AGS monolayers and then incubated at 37°C for 3 and 6 h. Culture media were gently removed and the infected monolayers were washed with PBS for four times, followed by a lyses procedure with distilled water for 10 min. The lysates were serially diluted in PBS, plated onto Dent blood agar plates and cultured for 4–5 days, after which the cfu were counted.

### Detection of IL-8

AGS cells (4×10^4^) grown in 48-well plates were infected with the wild-type or *ccrp* deletion mutant cells at an MOI of 100 for 24 h or in the case of the kinetics for hours as indicated. The amount of IL-8 secreted into cell culture supernatants was determined by ELISA by using the IL-8 Eli-Pair Kit (Diaclone) according to the manufacturer’s instructions.

### Immunoblotting

Equal amounts of total cell lysates were subjected to SDS-PAGE and transferred onto nitrocellulose membranes (Hybond Extra, GE Healthcare Life Sciences). Membranes were incubated with appropriately diluted primary antibodies at 4°C overnight and then probed with secondary antibodies conjugated with horseradish peroxidase. Immunodetection was performed by enhanced chemiluminescence Plus Western Blotting Detection Reagents (GE Healthcare Biosciences).

### CagA Translocation Assay

Translocation of CagA into AGS cells was analyzed by co-culturing *H*. *pylori* strains with AGS cells and detecting tyrosine phosphorylation of CagA, as previously described [42]. Briefly, *H*. *pylori* and AGS human gastric cells were co-cultured at a MOI of 100:1 for 4 h at 37°C in six-well plates. After infection time the cells were put on ice and washed with PBS. Then PBS containing protease and phosphatase inhibitors (1mM Na_3_VO_4_ (Sigma), 1mM PMSF (AppliChem), 1μM leupeptin (AppliChem), 1μM pepstatin (AppliChem)) was added and the cells were scraped off the six-well plates. After centrifugation the cell sediment was resuspended in PBS containing protease and phosphatase inhibitors. CagA translocation was assessed by separating the soluble fraction using SDS-PAGE and immunoblotting with an anti-phosphotyrosine antibody (PY99, Santa Cruz Biotechnology).

### *In vitro*-phosphorylation of CagA

The phosphorylation of CagA of *H*. *pylori* wild-type and *ccrp59* mutant cell lysates was performed as described previously [43,44] with some modifications. Briefly, 2 × 10^7^ AGS cells were lysed in 300 μl of ice-cold NP-40 buffer (20 mM Tris-HCl, pH 7.5, 150 mM NaCl, 0,25% (w/v) sodium deoxycholate, 1 mM Na_3_VO_4_, 1 mM EDTA, 1 mM PMSF, 1 μM leupeptin, 10 μM pepstatin, 1% Nonidet P-40 (v/v)). 1ml of OD_600_ 1 of *H*. *pylori* grown in BBF were centrifuged and lysed in 22.5 μl of the same buffer. A mixture of each lysate was incubated together with 5 μl 10 x phosphorylation reaction buffer (250 mM Tris-HCl, pH 7.2, 400 μM ATP, 62.5 mM MnCl_2_, 312.5 mM MgCl_2_, 625 μM Na_3_VO_4_) for 60 min on ice. The lysates were mixed with SDS-sample buffer and analysed by immunoblotting using an anti-phosphotyrosine antibody (PY99, Santa Cruz Biotechnology).

### Immunofluorescence

Immunofluorescence of *H*. *pylori* cells was performed as described earlier [45,46] with the following modifications: anti CagY antibody (1:10000) and anti CagT antibody (1:5000) were used as primary antibody, which were detected by the secondary antibody goat anti-rabbit Alexa Fluor 488 (Invitrogen) (1:100).

### Statistical analysis

The Student’s *t*-test was used to calculate the statistical significance of the experimental results between two groups (significance at *P* < 0.05 is depicted with one asterisk, *P* < 0.01 with two and *P* < 0.001 with three asterisks).

## Results

### Deletion of Ccrp proteins leads to a decrease in urease activity

**Fig 1:**
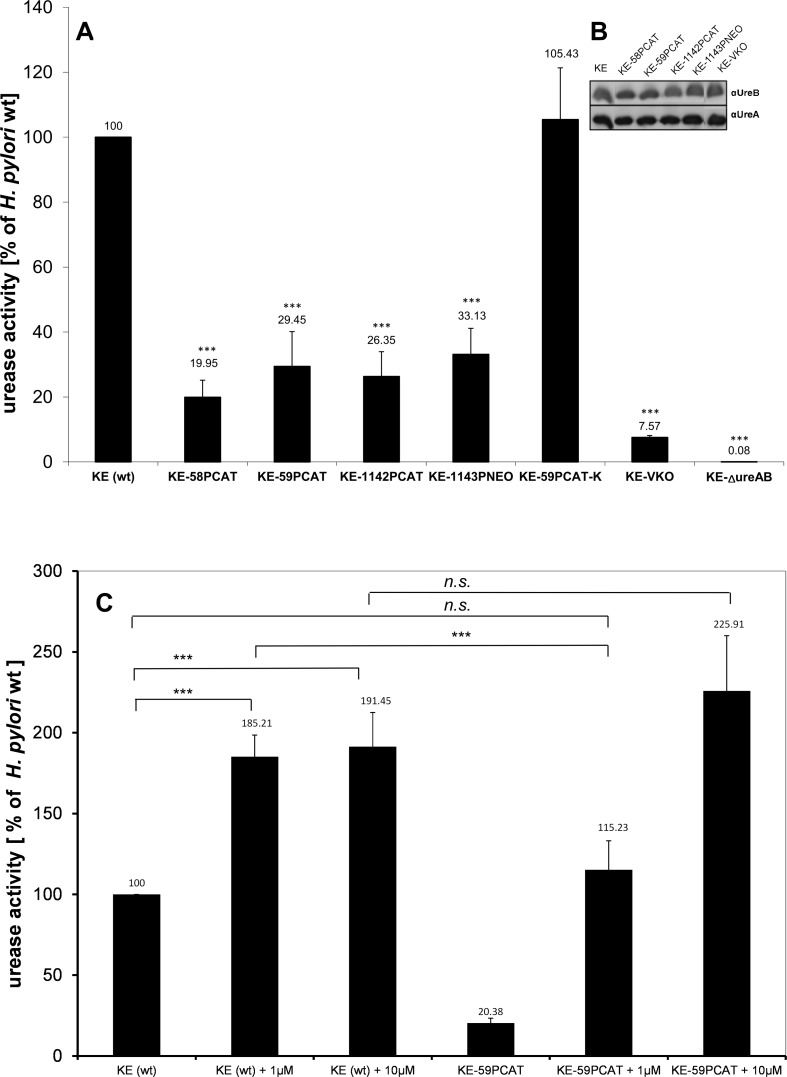
A) Urease activity of strain KE88-3887 wild-type and *ccrp* mutants. Activity is expressed as percentage relative to wild-type in unsupplemented medium (set at 100%; no error bar). Results shown are the averages of six independent experiments; error bars denote standard deviations. Exact percentage values are indicated above the bars. Asterisks indicate a significant difference in urease activity between *ccrp* mutants and wild-type *H*. *pylori* (the P value was <0.001, as determined by Student's t test). B) Western blot using urease specific antiserum and strains as indicated above the lanes. Equal amounts of protein were loaded onto each lane. C) Urease activity of the wild-type strain and the *ccrp59* mutant in unsupplemented and nickel-supplemented media (as indicated in μM) shown as a percentage relative to wild-type in unsupplemented medium (set at 100%; no error bar). Results shown are the averages of six independent growth experiments; error bars denote standard deviations. Exact percentage values are indicated above the bars. Asterisks indicate a significant difference in urease activity between ccrp mutants and wild-type *H*. *pylori* (the P value was <0.001, as determined by Student's t test).

**Fig 2:**
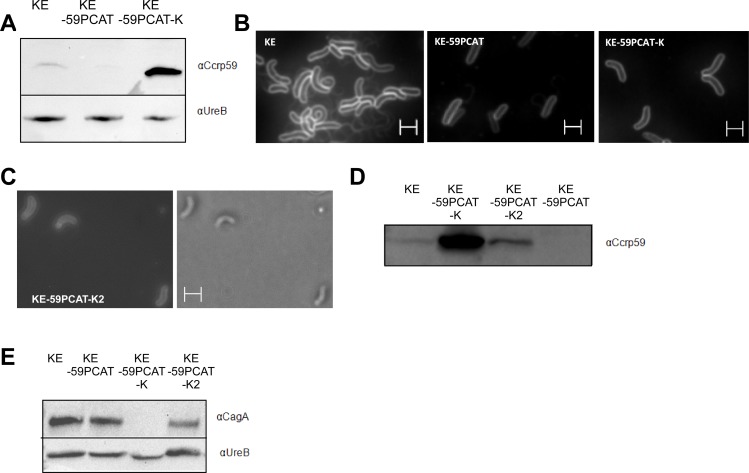
A) Western blot using Ccrp59 specific antiserum, strains as indicated above the lanes. Equal amounts of protein were loaded onto each lane. B) Fluorescent micrographs of FM4-64 membrane stained cells of KE88-3887 (KE) wild type, KE-59PCAT and KE-59PCAT-K. C) DIC and fluorescent micrographs of FM4-64 membrane stained cells KE-59PCAT-K2. D) Analysis of Ccrp59 production in KE88-3887 (KE) wild type, KE-59PCAT, KE-59PCAT-K and KE-59PCAT-K2 using Ccrp59 specific antiserum Equal amounts of protein were loaded onto each lane. E) Western blot analysis of CagA in KE88-3887 (KE) wild type, KE-59PCAT, KE-59PCAT-K and KE-59PCAT-K2. White scale bar 2 μm.

Previously we showed that loss of any Ccrp protein influences cellular processes like motility [29]. Here we set out to analyse whether the major pathogenicity factor urease is also affected by loss of Ccrp proteins. To this end, we determined urease activity in the different *ccrp* mutants of *H*. *pylori* strain KE88-3887 (KE) [28,29]. Interestingly, each *ccrp* deletion resulted in significantly reduced enzyme activity (p < 0.001) (Fig 1A): compared to the wild-type strain, *ccrp58*, *ccrp59*, *ccrp1142* and *ccrp1143* displayed only 19.95%, 29.45% 26.35% and 33.13% urease activity, respectively. Deletion of both *ccrp59* and *ccrp1143* resulted in further reduction to 23.8% of wild-type levels of urease activity (p < 0.001 in n = 8 separate experiments, data not shown), and deletion of all four *ccrp* genes led to a reduction to even 7.57% (Fig 1A). As a control, the urease activity of an *ureAB* deletion mutant was analyzed; this was zero as expected (Fig 1A). Furthermore, western blot analysis showed that the urease protein level in all *ccrp* mutant strains were indistinguishable from that in the parental wild type strain (Fig 1B). To evaluate whether the urease activity abrogated in the *ccrp59* mutant could be restored by introducing a full-length *ccrp59*, we generated a mutant in which an intact copy of *ccrp59* was integrated in KE-59PCAT. To this end, we used the strategy described in Schar *et al*. [36] and performed complementation by expression of the gene under the control of the ferritin promotor (*Ppfr*) at the unrelated *cagA* locus. In the resulting strain KE-59PCAT-K we then confirmed the presence of Ccrp59 via western blot (Fig 2A and 2D) and its functionality via analyzes of the cell morphology [28,29] (Fig 2B). The helical cell morphology was restored, but not pronounced (Fig 2B) despite a clear overproduction of Ccrp59 in KE-59PCAT-K (Fig 2A). Restoration resulted in an urease activity of 105.43% in KE-59PCAT-K (compared to wild-type level, Fig 1A). As the transition metal nickel (Ni^2+^) is an important co-factor of *H*. *pylori* urease, we also tested the influence of nickel addition on urease activity of the *ccrp59* mutant. Under *in vitro* growth conditions without additionally added Ni^2+^, only 2% of the active sites were filled with Ni^2+^ [47] Interestingly, addition of 1 μM NiCl_2_ restored urease activity up to wild-type level under unsupplemented conditions and addition of 10 μM NiCl_2_ resulted in in the loss of the significant difference of both urease activities (Fig 1C). In more specific terms, there was no significant difference by comparing urease activities of wild-type under unsupplemented conditions vs. *ccrp59* mutant under nickel supplementation of 1 μM indicating that the intracellular nickel concentration in the *ccrp59* mutant is reduced under unsupplemented conditions whereas nickel supplementation overcomes this effect. Based on this phenotype we can substantiate our previous results [28,29] that the Ccrp proteins of *H*. *pylori* are not only involved in the maintenance of cell shape but seem to directly or indirectly influence ion homeostasis and/or intracellular trafficking and thus also urease activity.

### Cytoskeletal mutants induce significantly fewer hummingbird AGS cells without reduced adhesion

**Fig 3:**
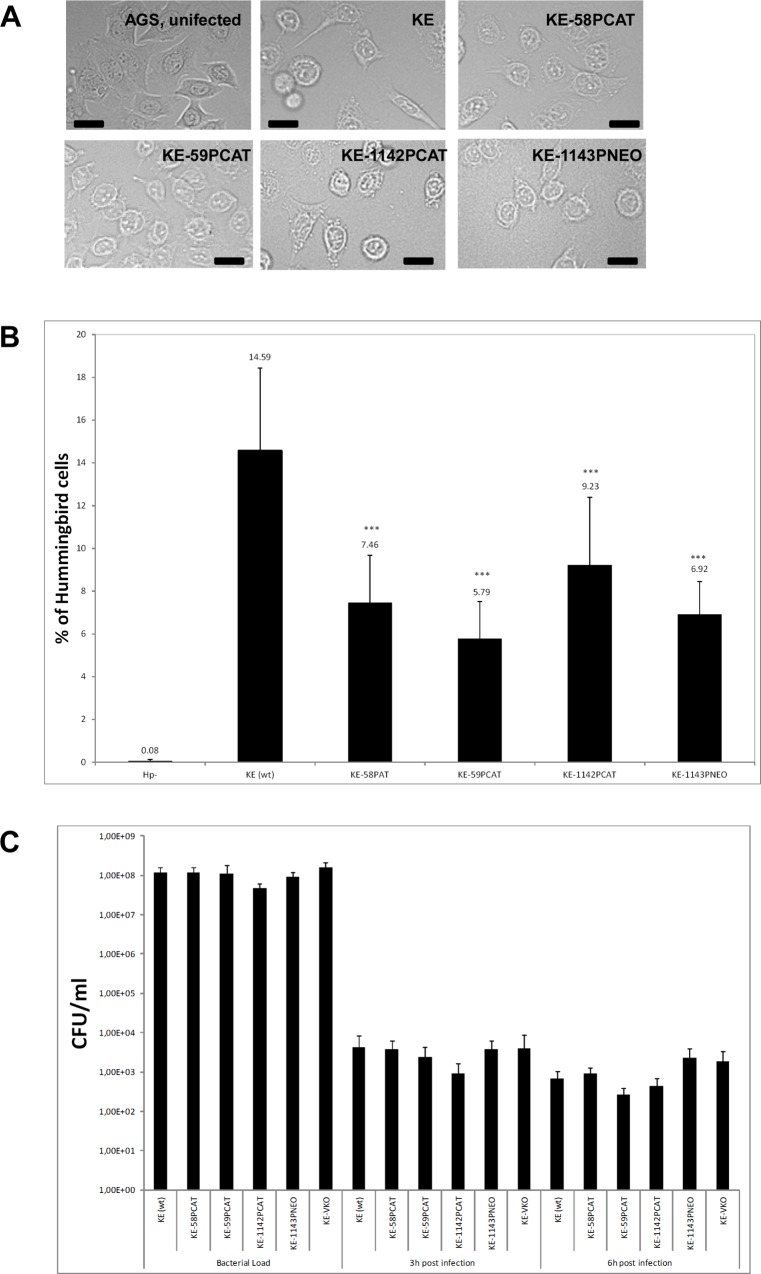
A) AGS cells co-incubated either with wild-type *H*. *pylori* cells (KE), *ccrp* deletion mutants as indicated or uninfected (AGS). Co-incubation was performed at MOI of 100 for 4 h. Cells were visualized by phase-contrast microscopy (BZ-9000E (KEYENCE) microscope) to assess AGS cell morphology. Scale bar, 20 μm. B) Quantification of the percentage of elongated cells from (A). All samples were examined in triplicate in at least three independent experiments. Data are presented as mean value of three independent experiments. For each strain between 1830 and 2800 cells were counted and evaluated. Exact percentage values are indicated above the bars. Asterisks indicate a significant difference between the *ccrp* mutants and wild-type *H*. *pylori* (the P value was <0.001, as determined by Student's t test). C) Bacterial adherence analysis in AGS cells infected with KE88-3887 or *ccrp* deletion mutants as indicated. AGS cells were infected with *H*. *pylori* for 3 h and 6 h, respectively. The number of cfu per cell was determined as described in experimental procedures and normalized to ml.

Since helical cell shape of *H*. *pylori* is an important pathogenicity factor we investigated whether lack of Ccrps influences bacterial virulence. To this end, we used *in vitro* co-culture experiments with the human gastric adenocarcinoma cell line AGS modeling *H*. *pylori—*host cell interactions. Infection of AGS cells with wild-type *H*. *pylori* causes a dramatic actin-dependent cell shape change known as the “scattering/hummingbird” phenotype, in which cells are transformed from a uniform polygonal shape into a severely elongated state characterized by the formation of needle-like projections [16,48]. Importantly, this phenotype is considered as a precursor of tumor development [49]. We analyzed the co-incubation of AGS cells with a MOI 100 (multiple of infection) of the wild-type strain KE or the different *ccrp* mutants derived from this wild-type strain [28,29]. First visual inspection of AGS cells gave the impression of a reduced “scattering/hummingbird” phenotype after co-incubation with *ccrp* deletion mutants (Fig 3A). Therefore we quantified our experiments by assessing up to 3500 cells of each strain per infection experiment. Quantification of the “hummingbird” phenotype of AGS cells after 4 hours of infection revealed that 14.59% AGS cells infected with wild-type *H*. *pylori* displayed the “hummingbird” phenotype (Fig 3B), whereas infection with any of the cytoskeletal mutants resulted in a clearly decreased proportion of “hummingbird” cells (Fig 3B). Deletion of all four *ccrp* led to no further reduction of the “hummingbird” phenotype (data not shown). We confirmed that our observed phenotypes were not caused by some secondary mutation or genomic change of single clones in the very variable organism *H*. *pylori* by repeating these experiments with independently derived secondary mutant clones of each mutant strain (S1 Fig). Furthermore a complemented strain of Ccrp59 was generated according to the strategy described in Croxen *et al*. [38] in which *ccrp59* was integrated with its own promoter into the *rdxA* locus. Functionality of the resulting strain KE-59PCAT-K2 as well as the presence of Ccrp59 and CagA were verified *via* cell shape analyses (Fig 2C) and western blot respectively (Fig 2D and 2E). By comparing the “hummingbird phenotype” of AGS cells after co-incubation of wild-type cells (100%) the KE-59PCAT-K2 complementation strain restored the phenotype to 91.19 +/- 1.5%.

Next, we analysed whether this observed reduced phenotype of AGS cells was caused by different adhesion abilities of the *ccrp* mutants to AGS cells. We performed *in vitro* bacterial adherence assays and analysed adherence of each strain by quantification of attached bacterial cells to up to 1100 AGS cells using immunofluorescence imaging. However, these experiments demonstrated that the adherence to AGS cells of wild-type *H*. *pylori* and mutant cells were comparable (data not shown) and thus could not be the reason for the reduced “hummingbird” phenotype. To confirm these results and to further clarify the viability of the adhered bacteria we used the standard colony-forming assay as previously described [41]. After 3 hours as well as after 6 hours there was similar viability during the infection process in the wild-type and all *ccrp* deletion mutants (Fig 3C). Consequently, these results demonstrate that deletion of any *ccrp* gene leads to a reduced “hummingbird” phenotype and that this phenotype is not caused by reduced binding ability to AGS cells or reduced viability during infection. Additionally these results also indicate that the previous observed reduced motility of these mutant strains [28] led not to a different adhesion behavior during co-incubation.

### *Ccrp59* deletion mutant induces less IL-8 production

**Fig 4:**
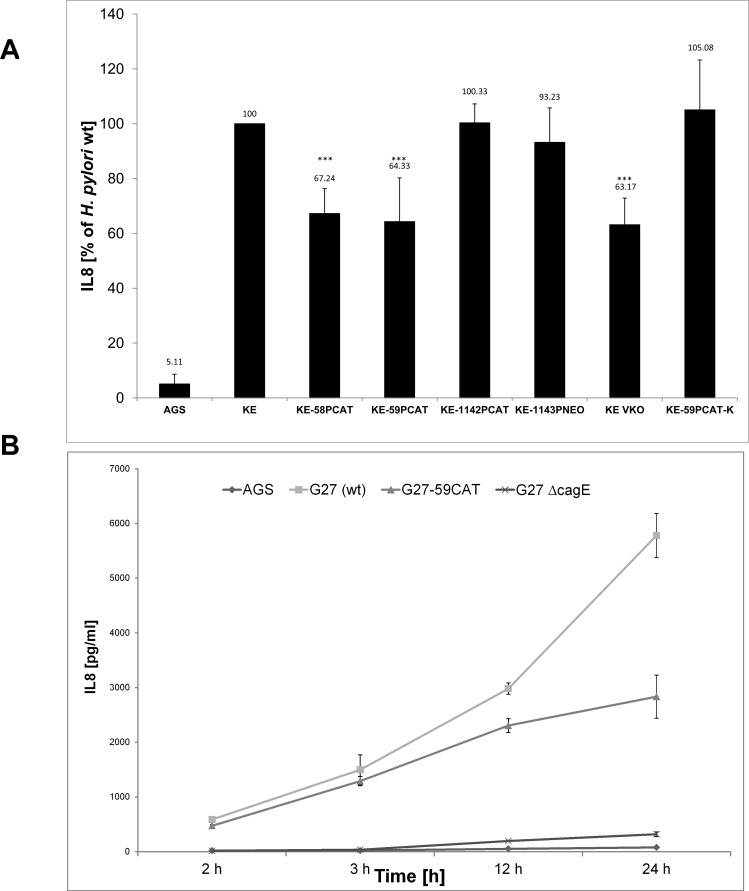
A) AGS cells were co-cultured with *H*. *pylori* wild type (KE) or the indicated mutants, and IL-8 production was analyzed by ELISA as described in Methods. Results shown are the averages of four independent experiments and expressed as a percentage relative to wild-type (set at 100%; no error bar); error bars denote standard deviations. Exact percentage values are indicated above the bars. Three asterisks indicate a significant difference with a P value below 0.001 as determined by Student's t test. B) Kinetics of IL-8 production shown as absolute values of *H*. *pylori* wild type (G27), *ccrp59* mutant (G27-CAT) and *cagE* mutant (G27ΔcagE), respectively; error bars denote standard deviations.

*H*. *pylori* interactions with gastric epithelial cells result *in vivo* in increased production of the proinflammatory chemokine interleukin 8 (IL-8), which is therefore often used as a marker for the inflammatory response of host cells. This induction has been proposed to be caused by the *cag*-T4SS mediated delivery of peptidoglycan into host cells and subsequent activation of either Nod receptors [13] or toll like receptors and the adaptor molecule MyD88 [50]. On the other hand several lines of indirect evidence suggest that the *cag*-T4SS apparatus *per se* elicits host pro-inflammatory responses independently of its substrates [15]. However, the molecular mechanisms of *cag-*T4SS system assembly and activity remain unclear. As a next step, we examined the IL-8 induction of AGS cells after infection with either wild type or any *ccrp* mutant of strain KE. Interestingly, a statistically significant difference was found between wild type and both the *ccrp58* and the *ccrp59* mutant whereas there was no significant difference between wild type and the *ccrp1142* or the *ccrp1143* mutant, respectively (Fig 4A). Deletions of all four *ccrp* lead to IL-8 induction of AGS cells comparable to those observed for *ccrp58* and *ccrp59* mutants (Fig 4A). As a consequence we focused in further examination on the *ccrp59* deletion mutant which has the strongest impact on the “hummingbird” phenotype. E. g., we elucidated the effect of this mutation on the kinetics of IL-8 production. A statistically significant difference was found between wild-type and the *ccrp59* mutant at all time points analyzed (data not shown) Furthermore, we confirmed the differences in IL-8 release after infection in a different strain background using *H*. *pylori* strain G27 (Fig 4C), and we also compared the IL-8 release of the *ccrp59* deletion mutant of G27 with the IL-8 production of a G27 mutant in which the *cag-*T4SS structural component *cagE* [51] was deleted (Fig 4B). Taken together, the *ccrp59* mutant stimulated a reduced *cag-*T4SS-dependent inflammatory host cell response compared to the levels of the wild-type strains (G27, KE), but ranged between the levels induced by wild-type and *cagE* mutant. We also analyzed our prior constructed strain KE-59PCAT-K in which we had inserted an intact copy of *ccrp59* into the *cagA* locus. Interestingly, this resulted in restoration of the impaired IL-8 production indicating CagA independency in this respect (Fig 4A).

### *Ccrp 59* deletion led to a reduced CagA translocation

**Fig 5:**
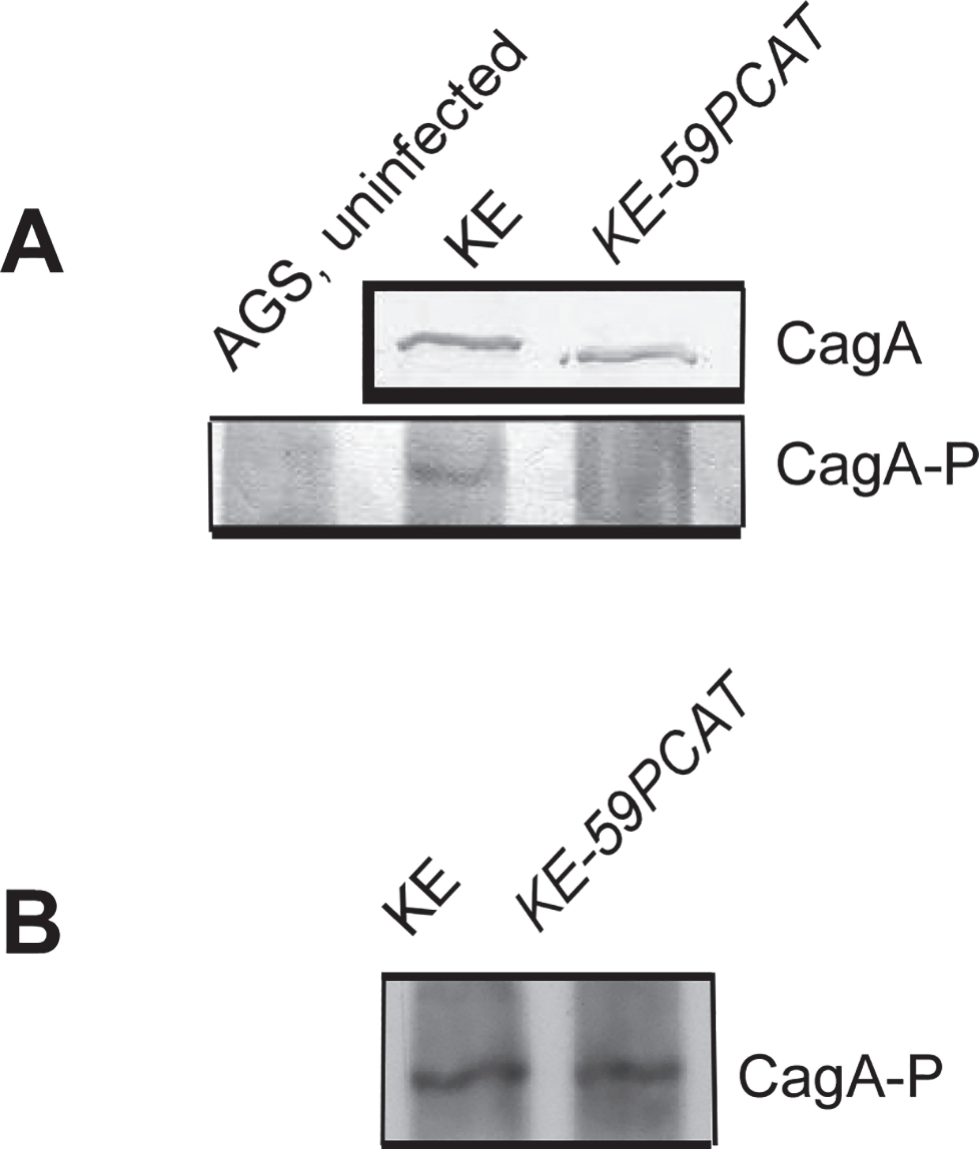
A) Analysis of CagA expression and CagA tyrosine phosphorylation in the *ccrp59* mutant. Bacterial lysates from *H*. *pylori* wild type KE and *ccrp59* mutant KE-59PCAT were prepared respectively. Each sample that consisted of equivalent amounts of protein was subjected to immunoblotting assay using antiserum against CagA. AGS cells were not infected or infected with these strains at an MOI of 100 for 4h and subjected to immunoblotting analysis using specific antibody against phosphorylated CagA (Cag-P). B) Control experiment showing that CagA of the *ccrp59* mutant can be phosphorylated in vitro by mixing *H*. *pylori* lysates with AGS cell lysates.

**Fig 6:**
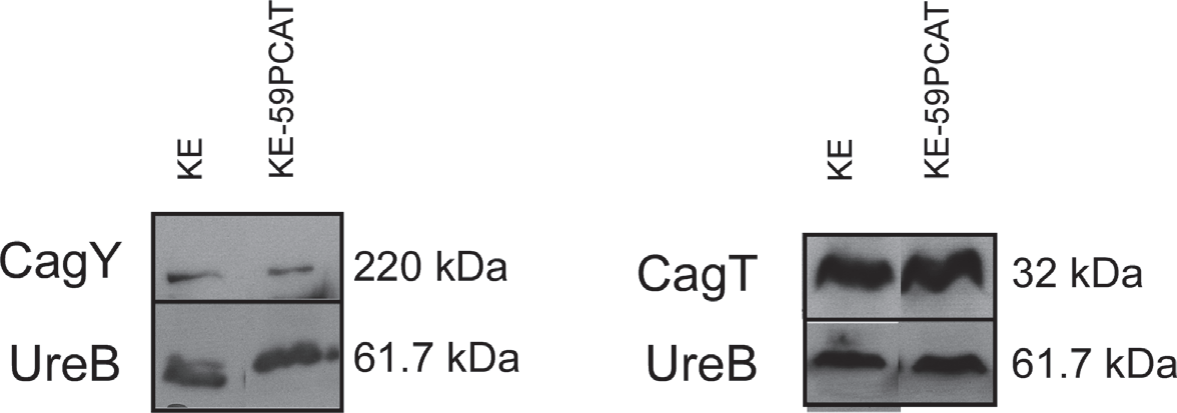
Western blot analysis of CagY and CagT in KE and KE-59PCAT respectively.

A prominent function of the *cag-*T4SS is the translocation of the effector CagA into gastric epithelial cells upon direct contact [12]. CagA is then tyrosine-phosphorylated (Cag-P) at a variable number of so-called EPIYA motifs by kinases of the Src and c-Abl family [48,52] and interacts with a large set of host proteins in phosphorylation-dependent and-independent ways. Also, CagA is regarded as a bacterial oncoprotein [17]. To assess the ability of the *ccrp59* mutant to translocate CagA into infected host cells, we determined CagA phosphorylation as a marker for its translocation [12],[53]. No, or very minor levels of, CagA phosphorylation were observed in KE-59PCAT infected AGS cells, although the *ccrp59* mutant expressed similar levels of CagA protein as wild type bacteria (Fig 5A). Calculation of the phosphorylation level of CagA in co-incubated AGS cells by densitometry quantification of the western blot bands of four independently derived experiments using the *ImageJ s*oftware revealed that compared to 100% wt density the *ccrp59* mutant displayed only 32 +/- 7%. In order to discriminate whether this lack of tyrosine phosphorylation indeed reflects a defect in translocation or whether the phosphorylation capacity was altered, we used an *in vitro* phosphorylation assay, in which the bacterial lysate is mixed with the epithelial cell lysate, thus circumventing the need to translocate the CagA protein before phosphorylation [43]. Both wild-type as well as *ccrp59* deletion mutant CagA protein were tyrosine-phosphorylated equally in this assay (Fig 5B). To address the question whether the *ccrp59* deletion affects *cag-*T4SS assembly we performed immunostaining experiments of *H*. *pylori* cells with antibodies against CagY and CagT. [54]. CagT is at the base of the *cag-*T4SS structure and is covered by CagY, a protein homologous to VirB10, that is part of the stable core complex spanning the cell membranes in *Agrobacterium* T4SS [55]. Together, CagT and CagY form the framework of the *cag-*T4SS [56]. Immunostaining experiments of the wild-type strain KE displayed a patchy pattern, reminiscent of local accumulation of CagY on the bacterial cell surface as published earlier [57]. However, comparison of CagY localization of the wild-type and the *ccrp59* mutant was not conclusive (data not shown). Very recently CagT localization has been observed in multiple clusters *via* immune gold electron microscopy suggesting a non-randomly distribution on the bacterial surface [58]. Whereas the clustered localization of CagT was confirmed for the wild-type strain KE in our experiments, the *ccrp59* mutant displayed a localization pattern with more CagT-clusters at the cell pole. Therefore we quantified the amount of CagT clusters at the cell pole in two independent experiments by assessing at least 400 cells per strain (Table 2). Whereas in the wild-type strain 13.0 +/- 3.5% CagT clusters were located at the cell pole, 28.5 +/- 5.9% cell pole located CagT clusters were found suggesting a displacement of CagT. Albeit the fluorescence signals for CagY were much weaker in the *ccrp59* mutant, western blot analysis showed that the protein levels of both CagT and CagY were comparable in the *ccrp59* mutant and in wild-type strain (Fig 6). This suggests *cag-*T4SS formation was not fully distorted in *ccrp59* deleted bacteria and supports the notion of an accessory, rather than a central structural function of Ccrp59 in the activation of the *H*. *pylori cag-*T4SS.

**Table 2:** Quantification of CagT localization in immunostaining micrographs.

	KE (wt)	KE-59PCAT
Number of cells counted	405	427
Percentage of polar CagT clusters	13 +/- 3.5	28.5 +/- 5.9

## Discussion

Bacterial cells possess a dynamic cytoskeleton composed of diverse classes of self-assembling polymeric proteins. As such cytoskeletal proteins are often involved in the subcellular organization of bacterial cells [59]. E.g., cytoskeletal proteins have already been observed to be essential for the polar localization of pili in *Pseudomonas aeruginosa* [60], and for motility as well as for Type 3 secretion in *Salmonella* [61]. Furthermore, recent research changed the notion of a structureless, homogeneous bacterial cytoplasm through which macromolecules diffuse freely interacting only after random collisions. Today, the bacterial cytoplasm is seen as a system of macromolecular machines designed for specific functions assembled at specific locations and appropriate time points [62]. In the present study we have extended our knowledge on the Ccrp cytoskeletal elements by analysing their influence on pathogenicity factors of *Helicobacter pylori*.

Various bacterial factors are involved in the strict host and tissue specificities exhibited by *H*. *pylori* [63]. Among them, urease helps to withstand the acidic pH surrounding the bacteria and allows their survival in the gastric environment [9]. Previously we have shown that *H*. *pylori* has a novel type of system for the establishment and maintenance of defined cell morphology, which also influenced motility suggesting further cellular functions [28,29]. [26]. This system is composed of four Ccrp proteins containing a molecular architecture which is reminiscent of that of intermediate filaments [30,31]. Deletion of *ccrp59* results in the complete loss of helical cell shape, while inactivation of other *ccrp* genes affects cell morphology to a lesser extent depending on the strain background [28,29]. Strikingly, here we show that deletion of any *ccrp* gene caused a statistically significant decrease in urease activity whereas the amount of urease subunits was unchanged. In addition, deletion of all four *ccrp* genes leads to further reduction of this enzyme activity suggesting a synergistic effect. This effect could be rescued by an increase in the concentration of nickel, which is a limiting factor for the enzyme [32]. Also, introducing a full-length copy of *ccrp59* under the control of the Pfr promoter restored the impaired phenotype. At present, we have no clear explanation as to how these cytoskeletal elements might affect the activity of this enzyme. Possibly, the loss of cytoskeletal elements disrupts the spatial organization of bacterial proteins inside the cell affecting, e.g., the activity of membrane proteins such as transporters thereby changing the intracellular levels of metals and ions.

*H*. *pylori* produces a number of important virulence factors inducing a local inflammation in the stomach. Among them is the *cag*-T4SS, which injects the effector protein CagA as well as peptidoglycan [13] into the host cell. T4SSs are multicomponent membrane-spanning transport systems ancestrally related to the conjugation processes, which can also fulfil diverse tasks such as DNA uptake and release, or translocation of effector into the target cell. Much of the biochemical work done to understand T4SS has used the prototypical VirB/D4 T4SS encoded in the T-DNA of the plant pathogen *Agrobacterium tumefaciens*. In this canonical T4SS, the core complex consists of the hub protein VirB10 inserting into both the inner and outer membranes and spanning the entire width of the periplasm, to which VirB7, a lipoprotein, and VirB9 are attached [55,64]. We here demonstrate for the first time a connection between cytoskeletal elements and the *H*. *pylori cag-*T4SS membrane-spanning complex. Extensive research has been conducted to analyze the architecture and function of the different components of the *H*. *pylori cag-*T4SS [55]. In this study, we examined whether lack of any Ccrp would influence bacterial virulence by using the human gastric adenocarcinoma cell line AGS as a host cell infection model of. A hallmark of *H*. *pylori*-infected AGS cells is the development of the so-called “hummingbird” phenotype which is fully dependent on the presence of injected CagA and a yet unknown structural or injected *cag-*T4SS-factor causing AGS cell motility [65]. Strikingly, deletion of any *ccrp* gene resulted in a reduced formation of “hummingbird cells” whereas adhesion to the AGS cells and viability of bacteria during infection was not affected. This reduced formation of “hummingbird” AGS cells was restored by introducing a full-length copy of *ccrp59* including its own promoter into the *rdxA* locus. Another feature of *H*. *pylori* infection is the increased production of IL-8, which has been proposed to be mediated by a still uncharacterized *cag-*T4SS mediated delivery of peptidoglycan into host cells [24]. Interestingly, a significantly lower secretion of IL-8 was observed in *ccrp58* and *ccrp59* mutants, whereas there was no difference between wild type and the *ccrp1142* and *ccrp1143* mutants. Closer examination of *ccrp59* mutant co-incubated AGS cells revealed that this difference remains over time both in strain KE and in strain G27 indicating general validity. However, the reduction of IL-8 release was not as great as upon deletion of the *cag-*T4SS structural component CagE [51], suggesting an accessory, rather than a central structural function of Ccrp59. We also analysed the translocation of the *H*. *pylori* CagA effector protein via the *cag-*T4SS into host cells during infection. Despite comparable levels of CagA expressed in *H*. *pylori* wild-type and *ccrp59* mutant cells, phosphorylation of CagA was clearly diminished following infection with the *ccrp59* mutant. The phosphorylation capacity of CagA itself was not altered indicating an impaired Cag A translocation in the *ccrp59* mutant. However, as all these efects were strongly reduced but not completely absent these results support the idea that the assembly of the *H*. *pylori cag*-T4SS system is somehow disordered in the *ccrp59* mutant. Likewise, the initiation of localization of the type IV pilus PilT in *Pseudomonas aeruginosa* has been shown to be dependent on the cytoskleletal element MreB, and a yet unknown factor was claimed to be responsible for maintenance of this localization pattern [60]. Furthermore, recent analyses of the spatial distribution of fluorescence-labeled T4SS components of the *A*. *tumefaciens* VirB/D4 T4SS using quantitative image analysis and modelling revealed that T4SS foci were found to localize on the bacteria cell surface in a non-random periodic pattern [66]. Thus Cameron *et al*. stated that one possible explanation of this finding might be that the T4SS complexes might interact directly with a helical scaffold. As such, T4SS complexes might be positioned by a cytoskeletal scaffold or an interaction between T4SS assembly and a separate process (such as cell growth) that results in a periodic assembly pattern [66]. Determining the influence of Ccrps on subcellular localization of *cag-*T4SS is complicated by the presence of *cag-*T4SS proteins in multiple subcellular sites and the lack of clear localization data of the *cag-*T4SS, e.g., functional fluorescent protein fusions inside of *H*. *pylori* cells. Furthermore our knowledge about virulence factor localization inside of *H*. *pylori* cells is still very marginal. Factors involved in pilus assembly need to be present at the right time, in the right amount, and in the right place. In support of this idea, we analyzed the influence of *ccrp59* deletion on CagY and CagT *via* immunostaining and western blot analyzes. CagT, a VirB7 homologue, is located at the pilus site and proposed to form an oligomeric ring-like-structure around the base of the pilus assembly [56]. Very recent data demonstrated *via* immuno gold electron microscopy that CagT localizes in multiple clusters suggesting a non-random distribution on the bacterial surface [58]. CagY, a protein partly homologous to VirB10 [56], is found to have several unusual features. It is thought to mediate contact between the inner and outer bacterial membrane [54], similar to what has been described in *A*. *tumefaciens* and other Gram-negative bacteria [55]. Additionally CagY has been shown to be surface-associated, and it was suggested that the *cag-*T4SS pilus is decorated locally or entirely by CagY [54,56],[67]. Furthermore, CagY is proposed to bind the *cag-*T4SS-specific host cell receptor β1α5 integrin [68,69]. Using immunostaining, we confirmed the patchy localization of CagT in wild-type strain KE. Interestingly, the distribution seen in the *ccrp59* mutant was twice as much pronounced at the cell pole which suggests an influence of Ccrp59 on CagT localization. It was not possible to determine any difference in localization pattern of CagY as the immunostaining pattern itself was not conclusive. Additionally, as no quantitative image analyzes of CagY localization are available so far, any statement about distributed localization patterns of this protein would not be reliable. Furthermore, immunostaining procedures could have drastic effects on protein localization compared to the distribution in living cells and it has been suggested in the literature to complement these data with live-cell imaging [70] which will be subject of further studies. Although induction of the T4SS pilus structure itself is reported to be contact dependent [56],[22,42], subassemblies prior to host cell contact of the T4SS have been reported as well [42], and it has been hypothesized that there could be a two-step process leading to CagA translocation from the bacterial cytoplasm into host cell as CagA has also been found on the bacterial surface in the absence of host cells [71]. As such, it is also possible that Ccrp59 is needed for formation of the whole pilus structure rather than for building subassemblies prior to host cell contact. To date, biogenesis of the *cag-*T4SS pilus structure and its role in CagA translocation mechanism are not well defined. It will be important to generate quantitative image analysis to further elicit this subject.

Taken together, our results show that Ccrp proteins influence many cellular processes and are thereby crucial for the virulence of *H*. *pylori* not only by maintaining the helical cell shape [28], but also for generating effective enzyme activity of the main pathogenicity factor urease and in case of Ccrp59 also for efficient *cag-*T4SS-associated activity possibly by acting as an underlying scaffold.

## Supporting Information

S1 Fig Comparison of the “hummingbird” phenotype reduction of two independently derived *ccrp* deletion mutant clones. (TIF)
