# Supplementary figures and images for: Coiled Coil Rich Proteins (Ccrp) Influence Molecular Pathogenicity of Helicobacter pylori
Source: PLoS One. 2015 Mar 30;10(3):e0121463. doi: 10.1371/journal.pone.0121463 (PMC4379086; doi:10.1371/journal.pone.0121463)

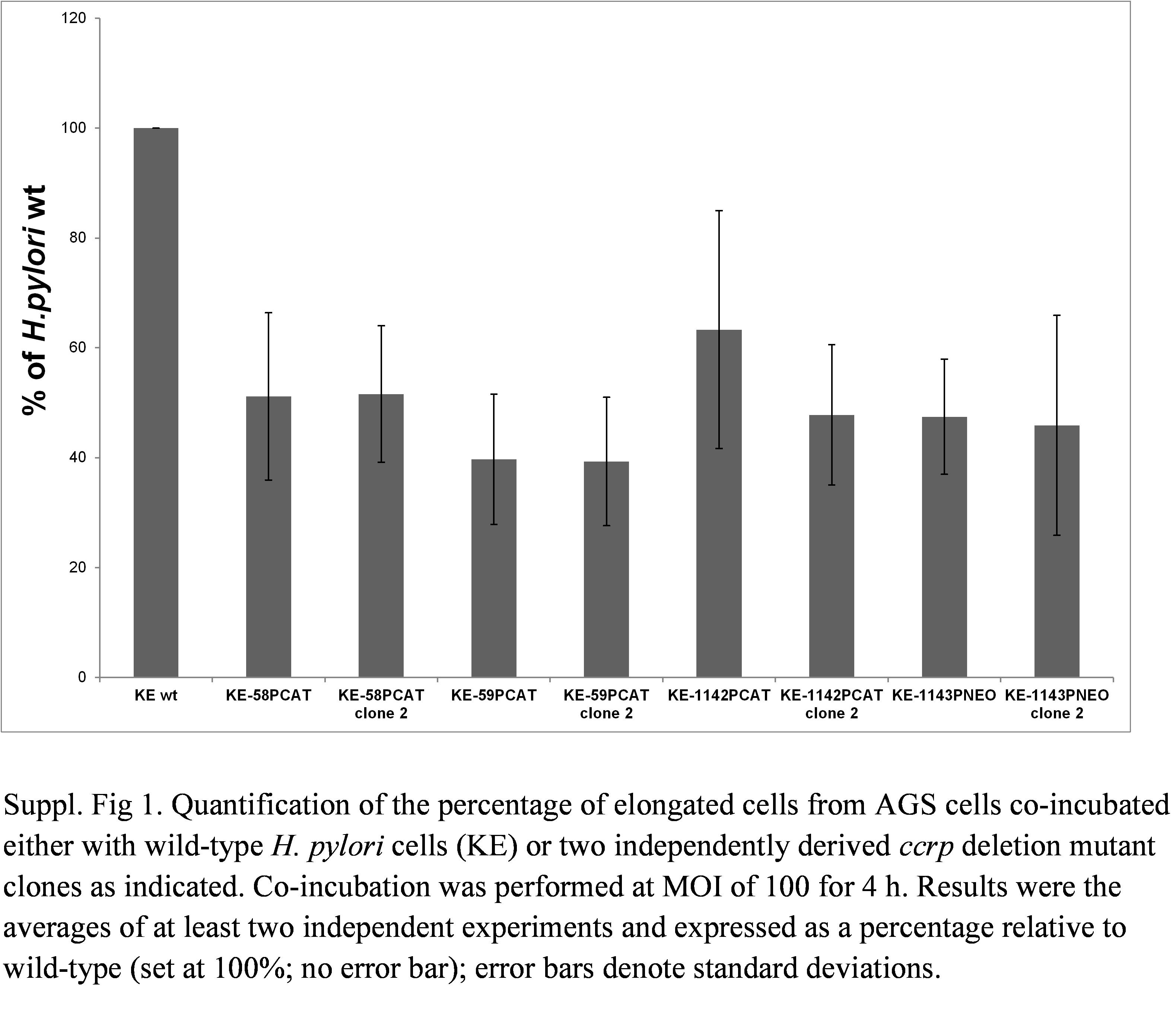

Supplement: S1 Fig — (TIF) [file pone.0121463.s001.tif]
